# Characterization of erythrose reductase from *Yarrowia lipolytica* and its influence on erythritol synthesis

**DOI:** 10.1186/s12934-017-0733-6

**Published:** 2017-07-11

**Authors:** Tomasz Janek, Adam Dobrowolski, Anna Biegalska, Aleksandra M. Mirończuk

**Affiliations:** 1 Department of Inorganic Chemistry, Faculty of Pharmacy, Wroclaw Medical University, Borowska 211a, 50-556 Wroclaw, Poland; 2 Department of Biotechnology and Food Microbiology, Wroclaw University of Environmental and Life Sciences, Chełmońskiego 37, 51-630 Wrocław, Poland

**Keywords:** *Yarrowia lipolytica*, Erythrose reductase, Erythritol, Glycerol

## Abstract

**Background:**

Erythritol is a natural sweetener that is used in the food industry. It is produced as an osmoprotectant by bacteria and yeast. Due to its chemical properties, it does not change the insulin level in the blood, and therefore it can be safely used by diabetics. Previously, it has been shown that erythrose reductase (ER), which catalyzes the final step, plays a crucial role in erythritol synthesis. ER reduces erythrose to erythritol with NAD(P)H as a cofactor. Despite many studies on erythritol synthesis by *Yarrowia lipolytica*, the enzymes involved in this metabolic pathway have ever been described.

**Results:**

The gene *YALI0F18590*g encoding the predicted erythrose reductase from *Y. lipolytica* was overexpressed, and its influence on erythritol synthesis was studied. The amino acid sequence of the *Y. lipolytica* ER showed a high degree of similarity to the previously described erythrose reductases from known erythritol producers, such as *Candida magnoliae* and *Moniliella megachiliensis*. Here, we found that the gene overexpression results in an enhanced titer of erythritol of 44.44 g/L (20% over the control), a yield of 0.44 g/g and productivity of 0.77 g/L/h. Moreover, on purification and characterization of the enzyme we found that it displays the highest activity at 37 °C and pH 3.0. The effects of various metal ions (Zn^2+^, Cu^2+^, Mn^2+^, Fe^2+^) on erythrose reductase were investigated. The addition of Zn^2+^ ions at 0.25 mM had a positive effect on the activity of erythrose reductase from *Y. lipolytica*, as well as on the erythritol production.

**Conclusions:**

In this study we identified, overexpressed and characterized a native erythrose reductase in *Y. lipolytica*. Further optimizations of this strain via metabolic pathway engineering and media optimization strategies enabled 54 g/L to be produced in a shake-flask experiment. To date, this is the first reported study employing metabolic engineering of the native gene involved in the erythritol pathway to result in a high titer of the polyol. Moreover, it indicates the importance of environmental conditions for genetic targets in metabolic engineering.

**Electronic supplementary material:**

The online version of this article (doi:10.1186/s12934-017-0733-6) contains supplementary material, which is available to authorized users.

## Background

Erythritol is a natural four-carbon polyol which occurs in wine, honey and fermented food. Due to its sweetness (70% of the sweetness of sucrose), erythritol has been used as a low-calorie sweetener for decades [1]. In humans, erythritol is not metabolized, but excreted by renal processes. Consequently, it has very limited potential to induce changes in insulin levels, and therefore it can be consumed by diabetics [2]. Its maximum no-effect dose for causing diarrhea is the highest among polyols, that is why it does not cause gastrointestinal side effects [3]. Erythritol can be synthesized from dialdehyde starch by a high-temperature chemical reaction in the presence of a metal catalyst, but this chemical process involves several steps, and therefore production costs are high [4]. For these reasons, on the industrial scale erythritol has been produced from glucose in biotechnological processes by osmophilic yeast such as *Trichosporonoides* sp. [5], *Candida magnoliae* [6] or *Moniliella* sp. [7]. It was shown that erythritol is also produced by the yeast *Yarrowia lipolytica* [8]. *Y. lipolytica* has been granted “generally recognized as safe” (GRAS) status [1]; therefore it is a safe producer of many proteins and other compounds such as polyols, lipids and organic acids [9–15]. This oleaginous yeast is able to utilize many unspecific carbon sources such as fatty acids, alkanes or crude glycerol [16]. Crude glycerol, the main co-product of diesel production, contains many undesired contaminants such as methanol, salts or heavy metals; hence its market value is still relatively low [17]. However, despite the contaminations, *Y. lipolytica* can easily utilize this substrate [9].

In yeast erythritol is produced as an osmoprotectant via the pentose phosphate pathway (PPP). Recently was shown, that also in *Y. lipolytica* its occurs via PPP [18]. The final step is catalyzed by erythrose reductase (ER), which reduces erythrose to erythritol with concomitant NAD(P)H oxidation [3, 19]. Several studies have been conducted on ER derived from *Candida magnoliae* [20], *Moniliella megachiliensis* (*Trichosporonoides megachiliensis)* [21] and *Trichoderma reesei* [22]. Moreover, it was shown that ER is a crucial gene in erythritol synthesis [4]. Despite this fact, the role of this enzyme in erythritol synthesis in *Y. lipolytica* is still unknown.

In this study we identified by in silico analysis predicted *Y. lipolytica* erythrose reductase (hereafter referred to as YlER), then we overexpressed the protein in *Y. lipolytica* and tested its influence on erythritol synthesis. In this study, we used glycerol as the sole carbon source for erythritol synthesis by *Y. lipolytica*, as it is a low-cost substrate that is beneficial for the industry. Moreover, we constructed a fusion of the *YALI0F18590g* gene encoding the hypothetical YlER with a C-terminal histidine tag to purify and characterize in enzymatic assays the protein for better understanding of its function. Here we found that overexpression of *YALI0F18590g* results in enhanced erythritol synthesis. Moreover, Zn^2+^ was found to be an important element for activity of YlER.

## Methods

### Microorganisms, media and culture conditions

The *Y. lipolytica* strains used in this study were derived from the wild-type *Y. lipolytica* A101 [23] and MK1 [19]. All of the strains used in this study are listed in Table 1.

**Table 1:** Strains and plasmids used in this study

Strain	Genotype or plasmid	Source
*E. coli*		
DH5α	F^−^ endA1 glnV44 thi-1 recA1 relA1 gyrA96 deoR nupG Φ80dlacZΔM15 Δ(lacZYA-argF)U169, hsdR17(rK-mK+), λ–	[52]
DH5α	pAD-YlER	This study
DH5α	pAD-YlERhis	This study
*Y. lipolytica*		
AJD	*MATA*, A101: ura3-302	[29]
AMM	*MATA*, MK1: ura3-302	[29]
MK1	*MATA, UV*-*mutant*	[19]
AJD pAD- YlERhis	*MATA*, A101: ura3-302, pAD-YlER	This study
AMM pAD- YlER	*MATA*, A101: ura3-302, pADUTGut2	This study

*Escherichia coli* strains were cultivated in LB medium according to standard protocols [24]. Rich yeast extract peptone glucose (YPD) medium was used for the yeast inoculum preparation and protein overexpression and contained 1% (w/v) yeast extract, 1% (w/v) peptone and 2% (w/v) glucose.

During shake-flask experiments the cultures were grown in 0.3 l flasks containing 0.03 l medium on a rotary shaker (CERTOMAT IS, Sartorius Stedim Biotech) at 28 °C at 240 rpm. Erythritol synthesis was conducted in the following medium (g/L): 100 glycerol (Chempur, Poland), 2.3 (NH_4_)_2_SO_4_ (Chempur), 1 MgSO_4_ × 7H_2_O (Chempur), 0.23 KH_2_PO_4_ (Chempur), NaCl 26.4, 1 yeast extract (Merk, Germany) and 3 CaCO_3_, pH 3.0, supplemented with 0.25 mM ZnSO_4_ × 7H_2_O (Chempur), when required.

### Bioreactor studies

To prepare an inoculation culture for fermentation in a bioreactor, the cultures were grown in 0.3 L flasks (containing 0.1 L of YPD medium) on a shaker at 28 °C for 72 h at 140 rpm. Erythritol production was conducted in a medium consisting of (g/L): 150 glycerol, 2.3 (NH_4_)_2_SO_4_, 1 MgSO_4_ × 7H_2_O, 0.23 KH_2_PO_4_, NaCl 26.4, 1 yeast extract, 0.25 mM ZnSO_4_ × 7H_2_O, pH 3.0.

### Sequence analysis

The searches for protein sequence were performed with BLAST at the National Center for Biotechnology Information (http://blast.ncbi.nlm.nih.gov/Blast). The amino acid sequences of ER were aligned with aldo–keto reductase sequences using Clustal Omega software [25]. A phylogenetic tree was constructed with SeaView software using the neighbor-joining method [26].

### Cloning and transformation protocols

All restriction enzymes were purchased from FastDigest Thermo Scientific (USA), and all of the digestions were performed according to standard protocols. The PCR reactions were set up using recommended conditions and Phusion high-fidelity DNA polymerase (Thermo Scientific). The ligation reactions were performed for 10 min at room temperature using T4 DNA Ligase (Thermo Scientific). The gel extractions were performed using the Gel Out gel extraction kit purchased from A&A Biotechnology (Poland). The *E. coli* minipreps were performed using the Plasmid Mini Kit (A&A Biotechnology). Transformation of *E. coli* strains was performed using standard chemical protocols [24]. Genomic DNA (gDNA) was extracted from *Y. lipolytica* using the Genomic Mini AX Yeast Spin kit (A&A Biotechnology).

### Construction of plasmids

After amplification of *Y. lipolytica* erythrose reductase encoded by *YALI0F18590g* with primers YlER-AscI-F (5′–CATG**GCGCGCC**ATGGCAGGCGGACCCAC–3′) and YlER-PmlI-R (5′-GCG**CACGTG**ATTTAAATGCTAGCTTACTTCTTCTGCTCAGCAAGGTA–3′), the 1006 bp PCR fragment was digested with *AscI* and *PmlI* and cloned into the corresponding sites of pAD-UTGut1 [27] to yield pAD-YlER.

Next, primers YlER-AscI-F and YlER-his-R (5′-GCG**GCTAGC**TTAATGGTGAT GGTGATGGTGACGCGTCTTCTTCTGCTCAGCAAGGTA–3′) amplified YlER with a his-tag on the C terminal end (his-tag sequence underlined), yielding a 1016 bp PCR product. Consequently, the *GUT1* gene was gel-extracted from plasmid pADUTGut1 [27] with AscI/NheI, and the obtained PCR product was inserted into the appropriate sites, to produce pAD-YlERhis.

The plasmids were digested with *MssI* to create linear expression cassettes devoid of *E. coli* DNA and surrounded by *Y. lipolytica* rDNA for targeted integrations. *Y. lipolytica* was transformed according to the lithium acetate method described previously [28]. The transformants were plated out on selective media [29]. Auxotrophies were restored via excision using the Cre-lox recombinase system following transformation with the replicative plasmid pUB4-Cre1(JME547) [30].

### Expression and purification

First, the obtained transformants of *Y. lipolytica* AJD carrying the overexpression cassette YlER-his were grown in liquid YPD medium. Next, 4 × 100 mL of fresh YPD medium was inoculated to obtain starting OD_600_ at 0.4. Since YlER was cloned under the UAS1B16-TEF promoter, whose activity reached the highest level after 24 h of growth [31], growth of the strain was continued for the next 48 h. Then the recombinant strains were harvested by centrifugation at 4 °C for 10 min at 2500 rpm. After washing with phosphate-buffered saline (PBS, 10 mM phosphate, 150 mM NaCl, pH 7.4), harvested cells were resuspended in 5 vol of homogenization buffer containing 50 mM sodium phosphate buffer (pH 7.0), 300 mM NaCl and protease inhibitor cocktail (Sigma-Aldrich). The sample was sonicated on ice for 10 min and centrifuged for 30 min at 20,000 rpm at 4 °C. The his-tagged erythrose reductase proteins were purified by metal-chelated affinity chromatography using a 1 mL HisTALON Gravity Column (Clontech, USA) with 20 mM imidazole in the washing buffer and 150 mM imidazole in the elution buffer. The protein concentration was determined according to the Bradford method [32]. The purity of the final products was analyzed by SDS-PAGE and Western blotting with the anti-His (C-term)-AP antibody (R932-25, Invitrogen) according to standard protocols.

### Enzyme assay

The enzymatic activity was determined as reported previously [4] with modifications given below. The reaction was initiated by mixing 1000 μL of 50 mM glycine–HCl/citrate/phosphate/Tris–HCl buffer (pH 2.5–9.0) containing 12 mM d-erythrose and 4 mM NADH and 10 μL of the purified enzyme. The absorbance at 340 nm was monitored at 22 °C for 10 min. The influence of metal ions on the activity of erythrose reductase was studied by assaying the enzyme activity at different ZnSO_4_, CuSO_4_, MnSO_4_ and FeSO_4_ concentrations (from 0.05 to 0.5 mM) at 28 °C in 50 mM citrate buffer (pH 3.0). For the effect of temperature, the activity of erythrose reductase was determined at different temperatures at pH 3.0. Substrate screening was performed with 4 mM NADH and 12 mM of the substrates l-arabinose, d-erythrose, d-fructose, d-glucose and d-galactose at a temperature of 28 °C at pH 3.0. The average of three measurements for each sample was adopted. One unit of enzyme activity was defined as the amount of enzyme which produced 1 μmol of NAD^+^ per minute under the above conditions.

### Circular dichroism spectroscopy

Circular dichroism (CD) measurements were recorded on a Jasco model J-1500 spectropolarimeter equipped with a thermostated cell holder (JASCO, Tokyo, Japan). Experiments were performed in a quartz cell with a 5 mm path length over the range of 200–260 nm at various temperatures, pH and different concentrations of metal ions. During CD spectroscopy analysis, respective purified erythrose reductase (0.1 mg/mL) was resuspended in the corresponding buffer (pH 2.5–9.0) and analyzed at 28 °C. Next, the spectra were acquired at pH 3.0 and pH 7.5 with different temperatures (10–53 °C), respectively. CD spectra were collected with a data pitch of 0.1 nm, bandwidth of 2.0 nm and scanning speed of 50 nm/min. The spectra represent the average of 6–9 scans, and data were analyzed using the K2D3 method. The K2D3 web server is an online tool used to assess the secondary structural elements in the form of α-helices and β-strands from far-UV CD spectra ranging from 200 to 240 nm [33].

### Fluorescence measurements

A cary eclipse fluorescence spectrophotometer was used for fluorescence measurements with an excitation wavelength of 280 nm, and the fluorescence emission was recorded in the range of 300–400 nm. The excitation and emission slit width (each 5 nm) was kept constant for all experiments. Fluorescence quenching measurements were recorded for erythrose reductase (15 mM) titrated with increasing concentrations of ZnSO_4_, CuSO_4_, MnSO_4_ and FeSO_4_ (0–0.5 mM) at pH 3.0 and room temperature (22 °C). All spectroscopic experiments were repeated at least three times.

### Zeta-potential measurements

The measurements of the zeta-potential of the aqueous suspensions of erythrose reductase were carried out on a Malvern Zetasizer Nano-ZS analyzer (Malvern Instruments Ltd., Malvern, Worcestershire, UK). The pH titration was performed manually by starting with the native sample at pH 6.0, then changing the pH using 0.1 N HCl. Next a fresh sample was created and the pH was changed using 0.1 N NaOH. Three zeta potential measurements were made at each pH value, and the average value was reported.

## Results and discussion

### *Yarrowia lipolytica* gene identification

**Fig. 1:**
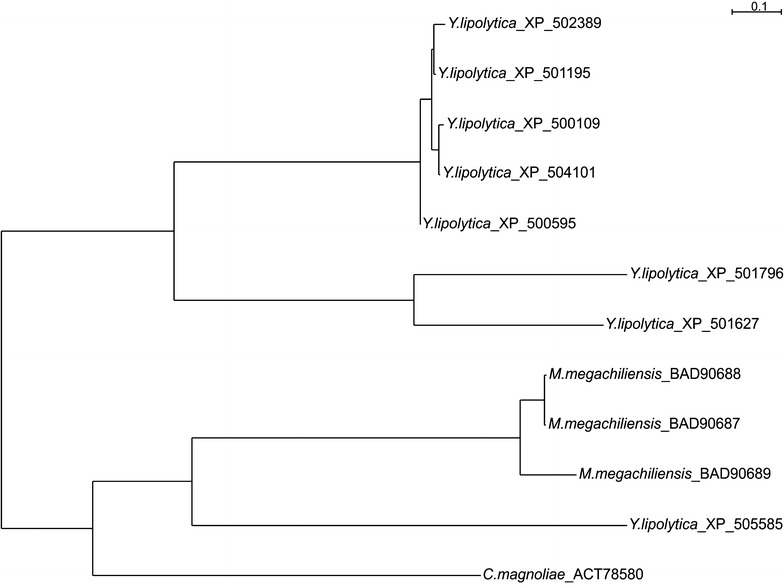
Phylogenetic analysis of the aldo–keto reductases from *Y. lipolytica*. The phylogenetic tree was constructed based on the alignment of full sequences of amino acids of known erythrose reductases. Proteins are identified by their GenBank accession number

The ability of *Y. lipolytica* to produce erythritol has been known for many years [8, 34]. However, for this species the metabolic pathway of this phenomenon has never been described. It was shown that one of the crucial enzymes in erythritol synthesis is erythrose reductase [4], which catalyzes the final step in this process. Up to now, the erythrose reductase from *Y. lipolytica* has not been characterized. Based on our previous study [19], we selected eight predicted proteins from the aldo–keto reductase (AKR) superfamily and compared them with other erythrose reductases available from the NCBI database using the BLAST program. Among the chosen proteins, the one (XP 505585) encoded by the *YALI0F18590g* gene was characterized by the highest homology to *C. magnoliae* ER (ACT78580 CmER) (41% identity) and to three erythrose reductases of *M. megachiliensis* (BAD90687 MmER1, BAD90688 MmER2, BAD90689 MmER3) with 44% identity (see Additional file 1: Table S1). As mentioned above, these species are known for their high erythritol synthesis, and their ERs have been well described. These in silico data suggest that the protein encoded by *YALI0F18590g* (YlER) might play a similar role in *Y. lipolytica*. To verify BLAST analysis, we constructed a phylogenetic tree with full length amino acid sequences of selected AKRs from *Y. lipolytica* and other erythrose reductases using Clustal Omega software (Fig. 1). As seen in Fig. 1, only YlER (XP 505585) was close to the CmER and all three isoenzymes from *M*. *megachiliensis.* Additionally, this protein was located far from other AKRs from *Y. lipolytica*, suggesting that it might have a different role in the metabolism. Moreover, there is a possibility that YlER evolved differently for the other AKRs from *Y. lipolytica*. Given this result, we overexpressed the predicted erythrose reductase in *Y. lipolytica* to verify its involvement in the erythritol pathway.

### Effect of YlER overexpression on erythritol synthesis

**Fig. 2:**
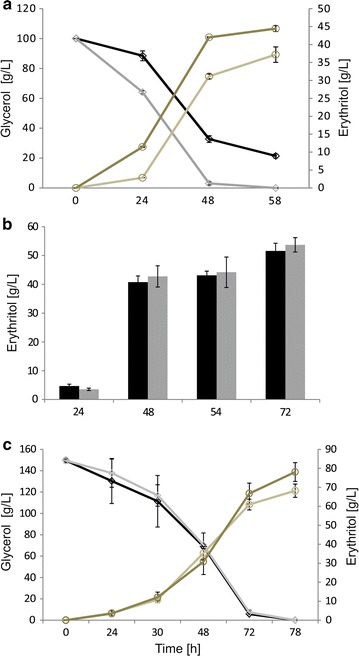
**a** Glycerol assimilation by AMM pAD-YlER (*gray*) and MK1 (*black*). Erythritol synthesis by AMM pAD-YlER (*dark brown*) and control strain (*light brown*); **b** erythritol synthesis in medium supplemented with 0.25 mM ZnSO_4_x7H_2_O by AMM pAD-YlER (*gray bars*) and control strain (*black bars*). The cultures were performed in triplicate. The *error bars* represent the standard deviation; **c** time courses of the 2 L scale batch fermentation in medium supplemented with 0.25 mM ZnSO_4_x7H_2_O glycerol assimilation by AMM pAD-YlER (*gray*) and control strain (*black*), erythritol synthesis by AMM pAD-YlER (*dark brown*) and control strain (*light brown*). The cultures were performed in triplicate. The *error bars* represent the standard deviation

The regulation and function of YlER protein is unknown in *Y. lipolytica*. Therefore, to verify the activity of all members of AKR family during erythritol synthesis, we compared the relative expression of the genes encoding the mentioned proteins. Remarkably, immediately after 24 h gene *YALI0F18590g* encoding YlER exhibited a 13-fold increase in expression (see Additional File 2: Figure S1A). Next, we overexpressed (see Additional File 2: Figure S1B) the predicted erythrose reductase to investigate whether the level of erythritol production was enhanced. For this purpose, the *YALI0F18590g* gene was cloned behind the constitutive UAS1B_16_-TEF promoter [31], the resulting plasmid pAD-YlER was digested, and consequently the overexpression cassette was introduced into the strain AMM. The strain AMM is derived from the well-characterized MK1 strain [19] that possesses natural ability for efficient production of erythritol from glycerol. It was found that production of erythritol occurs in high osmotic pressure conditions [35], so we performed a shake-flask experiment to verify the capacity of the engineered strain for erythritol synthesis and glycerol utilization. As a control, the parental strain MK1 was used. Interestingly, as seen in Fig. 2a, glycerol was depleted much faster by the engineered strain than by the control. The YlER overexpressing strain was able to utilize 100 g/L of glycerol within 58 h, whereas the control assimilated only 78.56 g/L. It was reported previously that overexpression of *GUT1* and *GUT2* genes (involved in assimilation of glycerol into the cell) results in enhanced utilization of glycerol [27]. Indeed we observed increased glycerol assimilation, whereas in this study the expression of *GUT1* and *GUT2* genes was not modified. However, the phenomenon of rapid glycerol assimilation was caused by pulling the metabolism toward erythritol synthesis [36] by overexpression of the last gene involved in this pathway. The biomass production for both of the strains remained at about the same level (12.7–12.85 g/L).

In agreement with our assumptions, the engineered strain produced more erythritol than the control (Fig. 2a). The strain overexpressing YlER produced 44.4 g/L of erythritol, whereas the control (MK1) produced only 37.1 g/L. In addition, the process parameters such as erythritol productivity (Q_ERY_) and yield (Y_ERY_) were significantly enhanced. AMM pAD-YlER achieved Q_ERY_ 0.77 g/L h, whereas MK1 achieved 0.64 g/L h; also Y_ERY_ was enhanced and it reached 0.44 g/g, while Y_ERY_ for the control was 0.37 g/g. This result is in agreement with a previous study, where the MK1 strain achieved 38.4 g/L of erythritol and Y_ERY_ was 0.39 g/g [19]. Interestingly, recently it was observed that some strains of the *Yarrowia* clade possess the capacity for erythritol production [37]. However, in that study the highest titer of erythritol obtained by *Yarrowia divulgata* and *Candida hollandica* in a shake-flask experiment was below 40 g/L. Moreover, the strain *Yarrowia lipolytica* A101 produced less than 25 g/L of erythritol during 6 days of cultivation. So far, this is the first reported study employing metabolic engineering of the native gene involved in the erythritol pathway to enhance the titer of the desired polyol. Recently a published work by Woude et al. described heterologous expression of various ER in the cyanobacterium *Synechocystis* [38]. In that study the maximum titer of erythritol was obtained by strain SEP024, which produced only 256 mg/L of erythritol.

Here, the elevated level of erythritol synthesis in the engineered strain suggests that the gene *YALI0F18590g* is involved in metabolism of this polyol. In this study, we observed that production of erythritol increased by 20%, but probably it could be even more elevated. As mentioned above, AKRs require NAD(P)H as a cofactor to catalyze the reaction. The main pool of NAD(P)H is produced via PPP, but in this study we did not overexpress genes involved in this pathway. For this reason, part of the overexpressed protein remained inactive due to titration loss of the available cofactor. The excess of protein is not functional when the concentration of cofactor is not sufficient. This issue needs further studies for a fuller understanding. As the research field is interesting, further studies will be conducted on co-expression in the near future in our laboratory.

Next, we deleted YALI0F18590g to check its influence on erythritol synthesis. Deletion of YlER results in lower erythritol production compared to the control, however, the process is maintained (see Additional File 3: Figure S2). Probably the role of the YlER was intercepted by the other protein homologue(s).

### Overexpression and purification of YlER

**Fig. 3:**
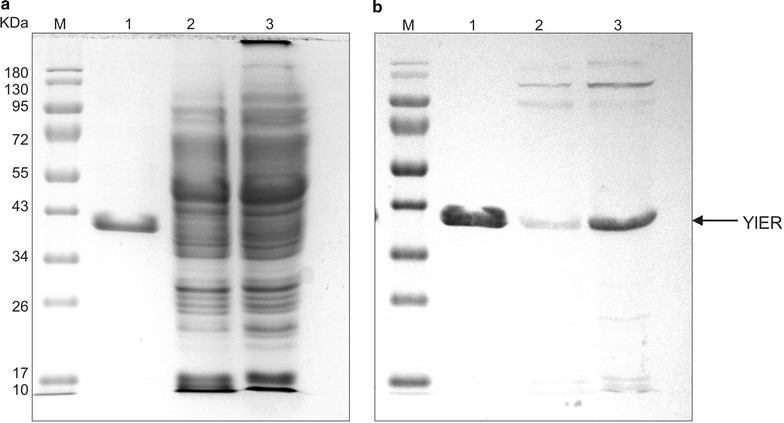
**a** SDS-PAGE and Western-Blot **b** analysis of YlER-his. *Lane M*—protein standard, *lane 1*—purified YlER protein; *lane 2*—wash II; *lane 3*—crude cell extract

The next aim of our study was to investigate the biochemical properties of YlER. To overexpress YlER in *Y. lipolytica*, a plasmid harboring a fusion of the C-terminus of YlER with a his-tag was transformed into the AJD strain, resulting in AJD pAD-YlERhis. In this experiment, we chose the AJD strain that is derived from the *Y. lipolytica* A101 strain, because it is characterized by high biomass production [29]. Next, the engineered strain was grown for 48 h in YPD medium to obtain a high yield of the protein. To verify the overexpression of YlERhis following UASB_16_-TEF activity, SDS-PAGE and Western blot analysis were performed using his-tag specific antibodies (Fig. 3). The final yield of YlER calculated from the optical density at λmax was 1.6 mg/L of *Y. lipolytica* cells with a molecular mass of ~37 kDa, which corresponds well to the calculated mass and the range of aldo–keto reductases [39]. Finally, the YlER showed that its purity was >98% (Fig. 3). The experiment above demonstrates that a reasonably high yield of UASB_16_-TEF-dependent overexpression of YlER can be attained in *Y. lipolytica*. The purified protein was used for the further analysis.

### Biochemical properties of YlER

**Fig. 4:**
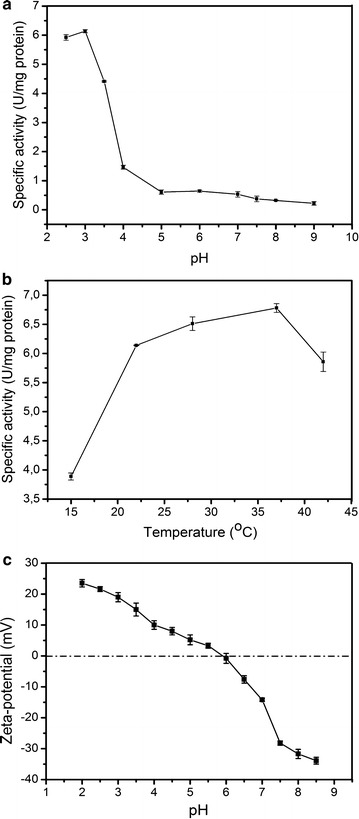
**a** Effect of pH on specific activity of YlER; **b** temperature dependence of YlER activity; **c** functions of zeta-potential on pH for aqueous suspensions of erythrose reductase. The analysis was performed in triplicate. The *error bars* represent the standard deviation

To examine substrate specificity, the protein activity was tested against various substrates. The results under standard assay conditions of pH 3.0 and 28 °C are presented in Table 2. The YlER was active with various aldose substrates. The enzyme showed a various specificity, with the highest specific activity against d-erythrose (6.51 U/mg), and lower specific activity against d-arabinose (6.25 U/mg) and d-galactose (6.11 U/mg). These obtained values were consistent with another characterized erythrose reductase enzyme from *C. magnoliae* [20] (Additional File 4: Table S2). To obtain more insight into the effect of pH and temperature on YlER activity we tested the activity of this protein in a wide range. First, the activity was tested at pH ranging from 2.5 to 9.0. As seen in Fig. 4a, the optimal pH for YlER activity is 3.0, at higher pH, its activity significantly decreases, and only about 15% of maximal activity was noted at pH above 4.0. This result explains the phenomenon of high titer of erythritol under acidic conditions. Previously, it has been observed that *Y. lipolytica* produces erythritol at low pH [8, 19] and during increasing pH of the environment the titer of erythritol decreases [40]. We suggest that this effect might be caused by inactivation of YlER. Higher pH of the environment results in carbon flux being pushed toward citric acid synthesis [12]. Given these results, we investigated the influence of temperature on YlER activity at pH 3.0. The maximum YlER activity was at 37 °C and slightly lower activity was noted at 28 °C. Below and above these temperatures the activity of YlER was significantly lower (Fig. 3b). Despite the fact that the optimal temperature for *Y. lipolytica* growth is 28–32 °C [1], YlER was the most active at 37 °C, this effect presumably being caused by higher kinetic energy of the molecules. Figure 4c presents the dependence of zeta-potential on pH for the analyzed YlER. At low pH, this protein presented positive zeta potentials, which decreased when pH was raised. The zeta-potential of YlER decreased from 23.5 mV at pH 2.5 to −33 mV at pH 8.5. The value of the isoelectric point (IEP), at which the zeta-potential of the enzyme was zero, was determined from Fig. 4c. The IEP of YlER was attained for pH 5.9. From theoretical calculations, the IEP of YlER was predicted to be 5.8. Our experimental IEP is slightly higher than the theoretical estimation, which can be explained by the fact that a small part of the charged amino acids is accessible to water.

**Table 2:** Substrate specificity of YlER

Substrate	Specific activity (U/mg protein)	Relative activity (%)
d-arabinose	6.25 ± 0.13	96
d-erythrose	6.51 ± 0.12	100
d-fructose	5.57 ± 0.04	86
d-galactose	6.11 ± 0.18	94
d-glucose	5.39 ± 0.25	83

### Circular dichroism spectroscopy analysis of YlER

**Fig. 5:**
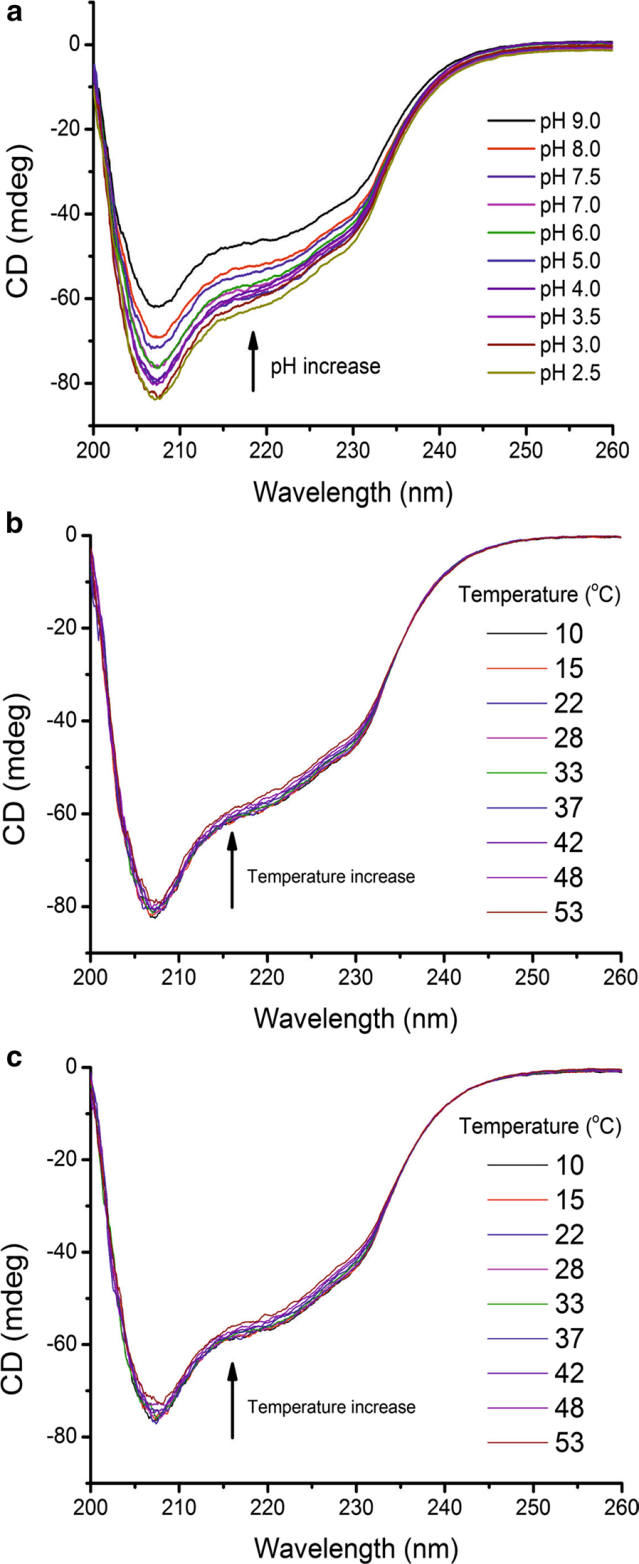
**a** Circular dichroism (CD) spectra of YlER at selected pH values and temperature 22 °C. Temperature dependence of circular dichroism (CD) spectra of YlER in 50 mM citrate/phosphate buffer at pH 3.0 (**b**) and pH 7.5 (**c**). The temperature ranges from 10 to 53 °C

Next, the secondary structure of YlER was investigated by circular dichroism (CD) spectroscopy. The CD spectrum was measured by monitoring the changes of the signal from 200 to 260 nm. The CD spectrum (Fig. 5a) showed the shape of the mainly *α*-helical secondary structure of the negative ellipticity bands near 207 nm. The CD spectrum of YlER is very similar in shape to that of *C. magnoliae* erythrose reductase [20] and human aldose reductase [41], indicating that all proteins have similar folding patterns. To estimate the secondary structure composition, the spectra (with different molar extinction coefficients, Δ*ε*) were analyzed by the K2D3 method [33]. Fitting the spectrum of native erythrose reductase at pH 3.0 and temperature 28 °C yielded a 45.3% *α*-helical structure. The *α*-helix content of YlER was more than that in human aldose reductase (40% *α*-helix, PDB accession code, 2R24) [42]. This difference might explain the extent of the YlER function compared to other aldose reductases. The effect of pH on the secondary structure of YlER was analyzed at 28 °C. Figure 5a shows the changes of secondary structure at each pH point comparing within a native sample at pH 7.0. The results show that the shape of the spectrum is changed, which could be interpreted as the occurrence of conformational modifications. The secondary structure of YlER reveals a decrease in *α*-helical content from pH 7.5 to pH 9.0. In contrast, the *α*-helical content was found to increase from pH 7.0 to pH 2.5 (Table 3). Regarding YlER activity as a function of pH, the enzyme showed the highest activity at pH 3.0 (Fig. 4a). Moreover, at pH 3.0 and temperature 28 °C YlER had the highest *α*-helix structure (Fig. 5a). To the best of our knowledge, *α*-helix structures are implicated in aldose reductases folding [43] as an appropriate structure for catalyzes the NADH-dependent conversion activity. Figure 5b presents the CD spectra of the YlER, at the temperature between 10 and 53 °C at pH 3.0 and pH 7.5 (B and C, respectively). Interestingly, in the case of YlER, all the CD spectroscopy curves at different temperatures showed a similar pattern at pH 3.0 (Fig. 5b) and pH 7.5 (Fig. 5c). Deconvolution of data between 200 and 260 nm indicated that the content of *α*-helices was stable over a wide temperature range. Increasing from 28 to 53 °C caused YlER to lose about 1% at pH 3.0 and 2% at pH 7.5 of the *α*-helix content, respectively. The effect of temperature on the secondary structure indicated that the secondary structure of YlER is not sensitive to the environment. In our opinion, the structure of YlER was stabilized by the hydrogen bond, electrostatic interaction and the hydrophobic effect.

**Table 3:** Contents of *α*-helix of erythrose reductase in the presence of various pH at 28 °C

pH	*α*-helix (%)	pH	*α*-helix (%)
2.5	47.6	6.0	44.3
3.0	47.4	7.0	47.7
3.5	45.4	7.5	45.4
4.0	45.3	8.0	42.6
5.0	45.8	9.0	36.5

### Effects of divalent metal ions on YlER activity and stability

Previously, it has been shown that minerals can bind directly to enzymes and enhance their activity. Motivated by the fact that mineral supplementation enhances erythritol synthesis by many various microorganisms [21, 44, 45] we set out to determine whether minerals have an effect on YlER activity. The optimum pH of 3.0 as the most favorable condition was selected for analyzing the effects of metal ions on YlER activity. As shown in Table 4, addition of Zn^2+^ increases the activity of this protein. The activity increases by 5% at lower Zn^2+^ concentration (0.05 mM) and 10% at higher Zn^2+^ concentration (0.25 mM) at 28 °C. Several other metal ions including Cu^2+^, Mn^2+^ and Fe^2+^ also considerably inhibited the enzyme. Collectively, these results suggest that the enzyme has a metal-binding site that it can accept a wide variety of divalent metal ions, modulating the activity of YlER.

**Table 4:** Effect of divalent metal ions on the activity of the purified YlER

Concentration (mM)	Relative activity (%)			
	ZnSO_4_	CuSO_4_	MnSO_4_	FeSO_4_
0	100 ± 0.2	100 ± 0.2	100 ± 0.2	100 ± 0.2
0.05	105 ± 0.6	99 ± 0.1	101 ± 0.5	102 ± 0.1
0.1	109 ± 0.1	98 ± 0.2	97 ± 0.3	93 ± 0.5
0.25	110 ± 0.3	96 ± 0.2	92 ± 0.4	90 ± 0.1
0.5	102 ± 0.4	95 ± 0.3	89 ± 0.3	89 ± 0.5

Data represent the means of three separate experiments

**Fig. 6:**
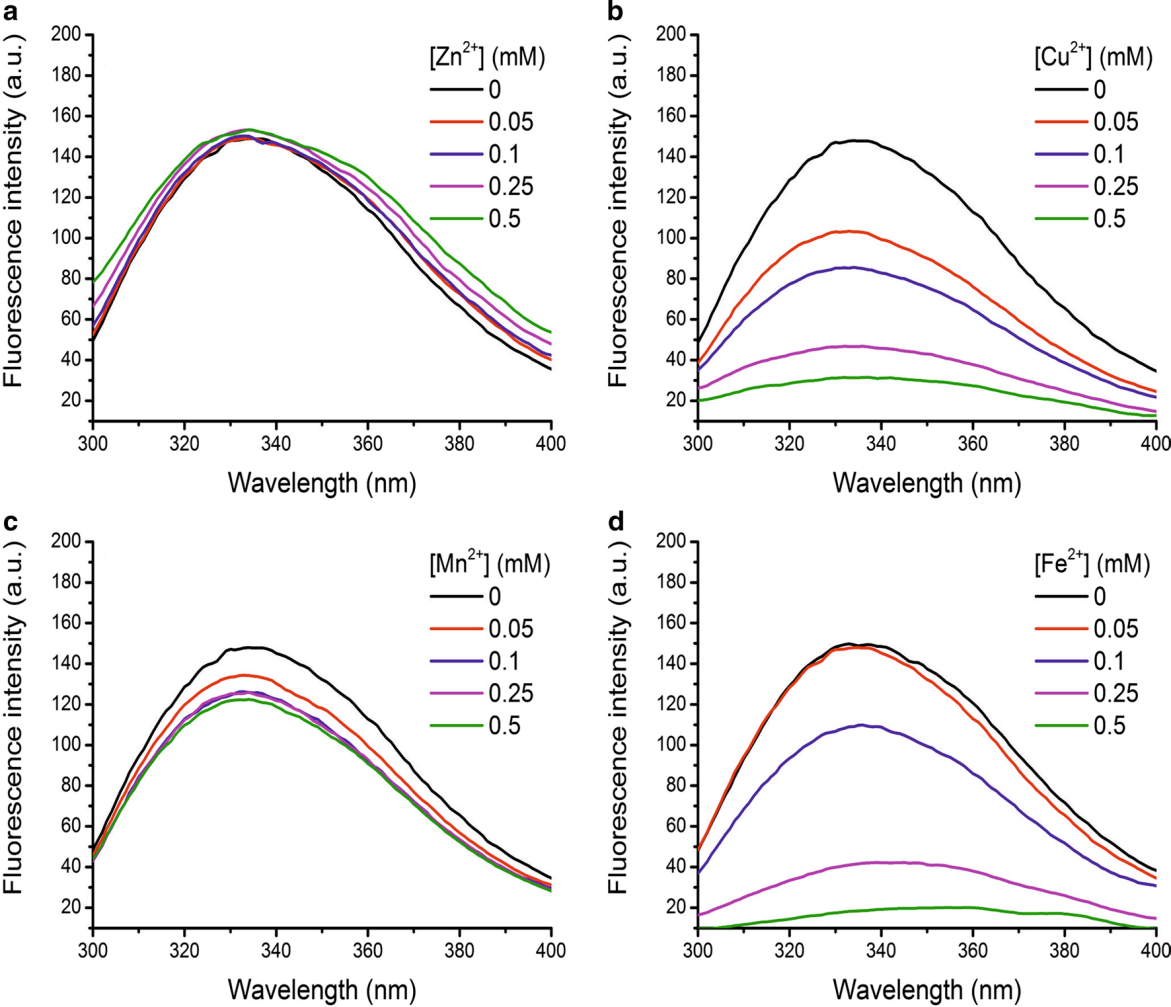
Fluorescence spectra of YlER in presence of increasing concentrations (0–0.5 mM) of Zn^2+^ (**a**), Cu^2+^ (**b**), Mn^2+^ (**c**) and Fe^2+^ (**d**)

**Fig. 7:**
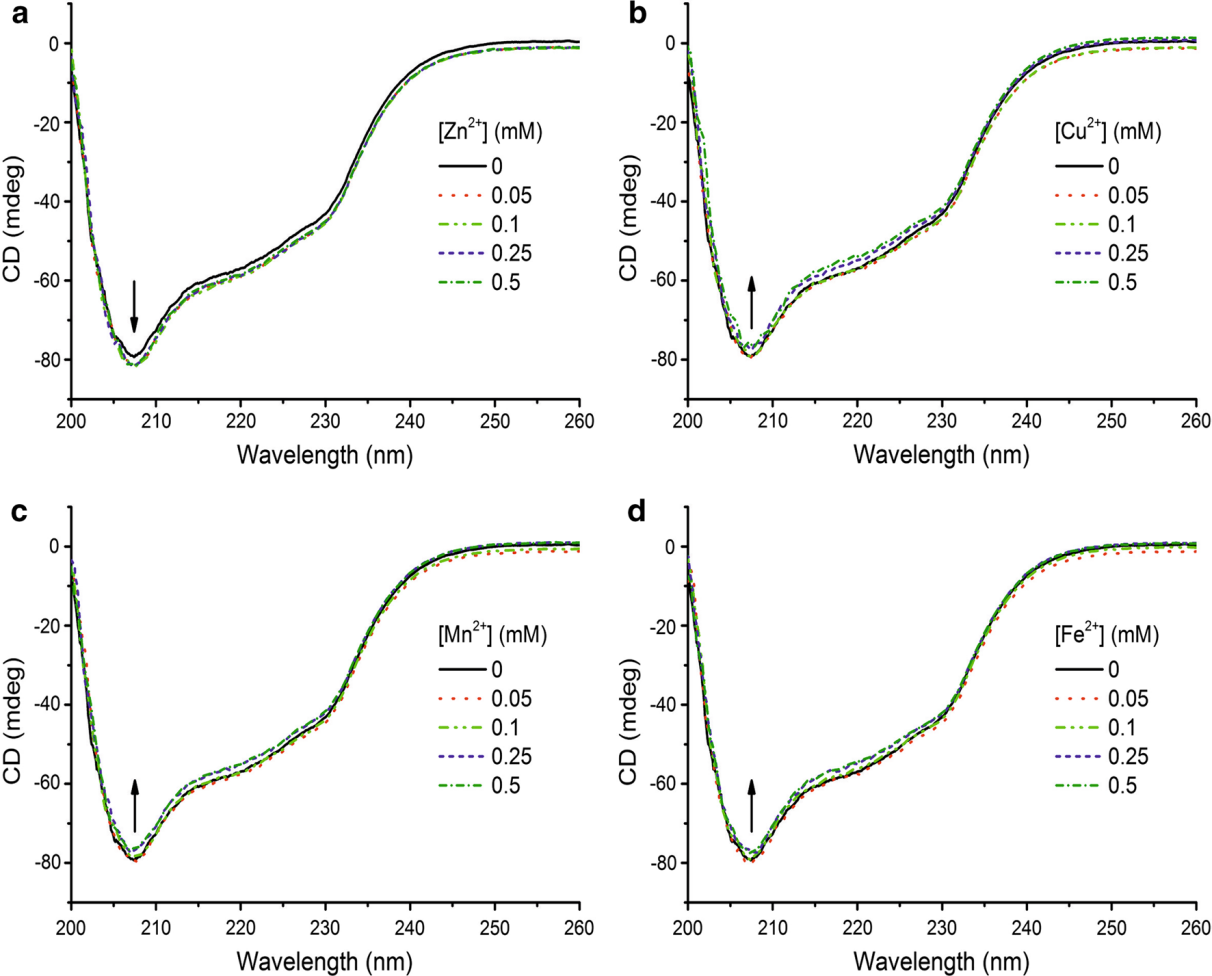
Circular dichroism (CD) spectra of YlER at different concentrations of Zn^2+^ (**a**), Cu^2+^ (**b**), Mn^2+^ (**c**) and Fe^2+^ (**d**) between 0.05 and 0.5 mM

Next, in our study, we employed a fluorescence spectroscopy to evaluate the integrity of the protein structure. As YlER contains tryptophan (Trp) and tyrosine (Tyr) residues, its fluorescence emission by excitation at 280 nm should be dominated by fluorescence of Trp residues due to the intramolecular energy transfer from Tyr to Trp residues [46]. To explore the metal ion-induced fluorescence change of YlER, fluorescence measurements were performed at an excitation wavelength of 280 nm. Interestingly, we observed that various metal ions have a different influence on the fluorescence intensity of this protein. As shown in Fig. 6, addition of Cu^2+^, Fe^2+^ or Mn^2+^ significantly decreases the fluorescence intensity of the protein. Metal ion-induced fluorescence quenching of YlER is attributed to the complex formation between protein and metal ions, and this binding perturbs the microenvironment around the Trp and Tyr residues and causes fluorescence quenching of the protein. Interestingly, the addition of increasing concentrations of Zn^2+^ to YlER showed a progressive fluorescence increase of the Trp donor, thus decreasing resonance energy transfer efficiency. The maximum excitation and emission of YlER were at 280 and 334 nm, respectively. No obvious shift of the maximum emission wavelength of YlER was observed, indicating that divalent metal ions have no significant influence on the polarity of the Trp residue. The previous experiment showed that the presence of divalent metal ions might have an influence on YlER; therefore to monitor the secondary structure changes of YlER under the same conditions the CD measurement was performed. The spectra were scanned over the wavelength range of 200–260 nm. Figure 7 presents the CD spectra of YlER with the divalent cations under study. With the increase of Zn^2+^, Cu^2+^, Mn^2+^ and Fe^2+^ concentrations, the shape of the spectrum changed, which might be interpreted by the occurrence of conformational modifications. Mn^2+^ binding induces small decreases in secondary structure content, but upon Cu^2+^ and Fe^2+^ binding the small decreases in *α*-helical content are larger (Table 5). On the other hand, Zn^2+^ binding has the opposite effect observed for Cu^2+^, Mn^2+^ and Fe^2+^, as it significantly increases the secondary structure of YlER. Interestingly, Zn^2+^ increases its *α*-helix content from 45.3 to 46.8%. Moreover, Zn^2+^ has also been reported to be the activator of several enzymes, including carbonyl reductase [47], xylanase [48], nattokinase [49] and lipase [50]. The tendency for a difference between Zn^2+^ and other metal ions may be due to the different structural compatibility of metal ions to bind with YlER.

**Table 5:** Contents of *α*-helix of erythrose reductase in the presence of various concentrations (mM) of metal ions in pH 3.0, at 28 °C

Zn^2+^		Cu^2+^		Mn^2+^		Fe^2+^	
(mM)	*α*-helix (%)	(mM)	*α*-helix (%)	(mM)	*α*-helix (%)	(mM)	*α*-helix (%)
0	45.3	0	45.3	0	45.3	0	45.3
0.05	46.2	0.05	44.9	0.05	45.8	0.05	45.0
0.1	46.4	0.1	44.7	0.1	45.2	0.1	44.5
0.25	46.8	0.25	44.1	0.25	44.2	0.25	44.2
0.5	46.8	0.5	42.2	0.5	43.9	0.5	42.3

### Effect of Zn^2+^ on erythritol production

Subsequently, we set out to determine whether Zn^2+^ supplementation increases erythritol synthesis by the engineered *Y. lipolytica*. In this experiment we compared AMM pAD-YlER strain harboring the overexpression cassette with its parental strain MK1. The strains were grown in baffled flasks in medium developed for erythritol synthesis, supplemented with 0.25 mM ZnSO4 × 7H_2_O. Interestingly, we noted a strong increase in erythritol titer for both of the strains, the engineered and the control strain (Fig. 2b). The erythritol titer was improved by 22 and 37% for the engineered (54.1 g/L) and the control strain (51.0 g/L), respectively, when compared to the results obtained in medium without zinc supplementation (Fig. 2a). In addition, for the engineered strain we noted an increase in Y_ERY_, which achieved 0.55 g/L/h. Interestingly, a significant difference was observed for the control, which achieved an erythritol titer (51.0 g/L) at a similar level to the engineered strain (44.4 g/L) in medium without supplementation. These results confirm that zinc has a great impact on production of this polyol by *Y. lipolytica*. Previously, it has been shown that zinc has a positive influence on erythritol synthesis [45]. However in that study, in medium supplemented with zinc the highest level of erythritol was 27.2 g/L. Probably the lower titer of polyol production was caused by lower osmotic pressure in the medium (medium without NaCl) and low rotation speed (140 rpm), which resulted in lower oxygenation of the medium. It has been noted before that different metal ions have a large impact on erythritol synthesis by various microorganisms [44, 51]. However, Zn^2+^ ions improved its production only in *C. magnoliae* and not in *Torula* sp. Moreover, the influence of metal ions on erythrose reductase activity has not been studied.

Next, to further characterize the engineered strain and explore its production abilities in medium supplemented with Zn^2+^ we performed large scale fermentation using a 5-L stirred-tank fermenter (Fig. 2c). Again, we used MK1 strain, as a control. In this experiment, the control strain produced 68.2 g/L of erythritol within 78 h of cultivation, with Q_ERY_ 0.87 ± 0.05 g/L/h and Y_ERY_ 0.45 ± 0.02 g/g. The strain overexpressing YlER produced 78.1 g/L of erythritol, and Q_ERY_ and Y_ERY_ were enhanced to 1.00 ± 0.12 g/L/h and 0.52 ± 0.06 g/g, respectively. Again, we observed enhanced erythritol synthesis by the engineered strain, nearly a 15% increase over the control bioreactor. It is important to note that also the process parameters (such as yield and productivity) were enhanced, what is beneficial for process development.

In summary, these experiments showed that Zn^2+^ has a positive influence on YlER activity and is an important factor in erythritol synthesis by *Y. lipolytica*. Moreover, it indicates the importance of environmental conditions on genetic targets for metabolic engineering.

## Conclusions

In this study we found that the predicted protein from the aldo–keto reductase (AKR) superfamily encoded by the *YALI0F18590g* gene is an erythrose reductase, which plays an important role in erythritol synthesis in *Y. lipolytica*. To our knowledge, this is the first reported efficient production of erythritol by genetically modified microorganisms by overexpression of the native ER. Moreover, the overexpression of YlER coupled with Zn^2+^ supplementation results in robust erythritol production from glycerol. This enzyme can be heterologously expressed in microorganisms which do not synthesize erythritol such as cyanobacteria. Further studies will focus on metabolic engineering, leading to an elevated NAD(P)H pool and optimization of culture conditions to improve the erythritol production capacities of *Y. lipolytica*.

## Additional files

**Additional file 1: Table S1.** Amino acid homology in ARK family.

**Additional file 2: Figure S1.** Quantification of genes expression belong to the ARK family, during erythritol synthesis (A). Quantification of *YALI0F18590g* gene expression (YlER,) by the strain AMM pAD-YIER and the control (B). Samples were analyzed in triplicate and the standard errors were estimated using Illumina Eco software. The results were normalized to actin gene ACT-F/ACT-R and analyzed using the ddCT method.

**Additional file 3: Figure S3.** Erythritol synthesis by strain AMM ΔYlER (gray bars) and control strain MK1 (black bars). The cultures were performed in triplicate. The error bars represent the standard deviation.

**Additional file 4: Table S2.** Relative activity (%) of the erythrose reductases from *Candida mangnoliae* (CmER) [20], *Yarrowia lipolytica* (YlER), *Moniliella megachiliensis* (ER-III) [21] and *Trichoderma reesei* (Err1) [22].

## Abbreviations

YlER: *Y. lipolytica* gene *YALI0F18590g*
ARK: aldo–keto reductase superfamily
ERY: erythritol
Q: productivity
Y: yield
