# Supplementary material for: Characterization of erythrose reductase from Yarrowia lipolytica and its influence on erythritol synthesis
Source: Microb Cell Fact. 2017 Jul 11;16:118. doi: 10.1186/s12934-017-0733-6 (PMC5504726; doi:10.1186/s12934-017-0733-6)
Supplement: Supplementary file 1 — Additional file 1: Table S1. Amino acid homology in ARK family. [file 12934_2017_733_MOESM1_ESM.doc]

**Characterization of erythrose reductase from *Yarrowia lipolytica* and its influence on erythritol synthesis**

**Tomasz Janek^1^, Adam Dobrowolski^2^, Anna Biegalska^2^, Aleksandra M. Mirończuk^2*^**

**^1^**Department of Inorganic Chemistry, Faculty of Pharmacy, Wroclaw Medical University, Borowska 211a, 50-556 Wroclaw, Poland

**^2^**Department of Biotechnology and Food Microbiology, Wroclaw University of Environmental and Life Sciences, Chełmońskiego 37, 51-630, Wrocław, Poland

*corresponding author [aleksandra.mironczuk@upwr.edu.pl](mailto:aleksandra.mironczuk@upwr.edu.pl)

Table S1. Amino acid homology in ARK family.

| **No** |  | Protein | **1** | **2** | **3** | **4** | **5** | **6** | **7** | **8** | **9** | **10** | **11** | **12** | **13** | **14** | **15** | **16** | **17** | **18** |
| --- | --- | --- | --- | --- | --- | --- | --- | --- | --- | --- | --- | --- | --- | --- | --- | --- | --- | --- | --- | --- |
| **1** | AKR1 | XP_501768.1 | 100 | 25.48 | 23.68 | 20.60 | 22.92 | 22.69 | 22.31 | 22.31 | 21.92 | 20.68 | 21.05 | 18.49 | 18.96 | 22.18 | 23.85 | 17.23 | 17.23 | 18.73 |
| **2** | AKR2 | XP_501280.1 | 25.48 | 100 | 63.48 | 31.95 | 29.78 | 31.48 | 31.11 | 30.74 | 30.74 | 28.73 | 32.00 | 28.47 | 35.19 | 30.47 | 33.72 | 27.37 | 27.37 | 27.01 |
| **3** | AKR3 | XP_500263.1 | 23.68 | 63.48 | 100 | 30.83 | 31.11 | 30.37 | 29.63 | 29.63 | 29.63 | 25.64 | 29.67 | 25.64 | 32.59 | 28.74 | 35.77 | 29.67 | 29.67 | 29.67 |
| **4** | AKR4 | XP_500922.1 | 20.60 | 31.95 | 30.83 | 100 | 32.98 | 31.91 | 31.91 | 31.91 | 31.56 | 32.37 | 32.97 | 30.53 | 32.85 | 36.11 | 36.30 | 32.75 | 32.75 | 31.36 |
| **5** | AKR5 | XP_500595 | 22.92 | 29.78 | 31.11 | 32.98 | 100 | 96.44 | 97.09 | 96.44 | 95.79 | 47.02 | 45.39 | 46.73 | 34.38 | 38.18 | 41.46 | 39.40 | 39.07 | 38.74 |
| **6** | AKR6 | XP_502389 | 22.69 | 31.48 | 30.37 | 31.91 | 96.44 | 100 | 97.41 | 96.44 | 95.15 | 45.67 | 45.70 | 45.57 | 33.68 | 38.35 | 40.91 | 38.54 | 38.21 | 38.21 |
| **7** | AKR7 | XP_501195 | 22.31 | 31.11 | 29.63 | 31.91 | 97.09 | 97.41 | 100 | 97.73 | 97.73 | 46.00 | 44.70 | 45.25 | 33.68 | 38.35 | 40.56 | 37.87 | 37.54 | 37.54 |
| **8** | AKR8 | XP_504101 | 22.31 | 30.74 | 29.63 | 31.91 | 96.44 | 96.44 | 97.73 | 100 | 98.71 | 46.00 | 45.03 | 45.25 | 33.68 | 38.72 | 40.56 | 38.21 | 37.87 | 37.87 |
| **9** | AKR9 | XP_500109 | 21.92 | 30.74 | 29.63 | 31.56 | 95.79 | 95.15 | 97.73 | 98.71 | 100 | 45.33 | 44.70 | 44.92 | 33.68 | 38.35 | 39.86 | 37.87 | 37.54 | 37.54 |
| **10** | AKR10 | XP_501627 | 20.68 | 28.73 | 25.64 | 32.37 | 47.02 | 45.67 | 46.00 | 46.00 | 45.33 | 100 | 56.77 | 53.40 | 30.93 | 36.09 | 34.62 | 36.75 | 36.75 | 35.43 |
| **11** | AKR11 | XP_501796 | 21.05 | 32.00 | 29.67 | 32.97 | 45.39 | 45.70 | 44.70 | 45.03 | 44.70 | 56.77 | 100 | 72.44 | 34.25 | 34.96 | 37.72 | 35.74 | 35.41 | 33.77 |
| **12** | AKR12 | XP_505102.1 | 18.49 | 28.47 | 25.64 | 30.53 | 46.73 | 45.57 | 45.25 | 45.25 | 44.92 | 53.40 | 72.44 | 100 | 32.19 | 33.96 | 36.33 | 35.35 | 35.35 | 32.28 |
| **13** | AKR13 | XP_502540.1 | 18.96 | 35.19 | 32.59 | 32.85 | 34.38 | 33.68 | 33.68 | 33.68 | 33.68 | 30.93 | 34.25 | 32.19 | 100 | 32.96 | 37.80 | 36.72 | 36.72 | 36.07 |
| **14** | C.magnoliae | ACT78580 | 22.18 | 30.47 | 28.74 | 36.11 | 38.18 | 38.35 | 38.35 | 38.72 | 38.35 | 36.09 | 34.96 | 33.96 | 32.96 | 100 | 40.15 | 43.43 | 43.07 | 40.88 |
| **15** | YlER | XP_505585 | 23.85 | 33.72 | 35.77 | 36.30 | 41.46 | 40.91 | 40.56 | 40.56 | 39.86 | 34.62 | 37.72 | 36.33 | 37.80 | 40.15 | 100 | 43.59 | 43.59 | 42.68 |
| **16** | M.megachiliensis | BAD90687 | 17.23 | 27.37 | 29.67 | 32.75 | 39.40 | 38.54 | 37.87 | 38.21 | 37.87 | 36.75 | 35.74 | 35.35 | 36.72 | 43.43 | 43.59 | 100 | 99.39 | 86.28 |
| **17** | M.megachiliensis | BAD90688 | 17.23 | 27.37 | 29.67 | 32.75 | 39.07 | 38.21 | 37.54 | 37.87 | 37.54 | 36.75 | 35.41 | 35.35 | 36.72 | 43.07 | 43.59 | 99.39 | 100 | 86.28 |
| **18** | M.megachiliensis | _BAD90689 | 18.73 | 27.01 | 29.67 | 31.36 | 38.74 | 38.21 | 37.54 | 37.87 | 37.54 | 35.43 | 33.77 | 32.28 | 36.07 | 40.88 | 42.68 | 86.28 | 86.28 | 100 |
