# Supplementary material for: Characterization of erythrose reductase from Yarrowia lipolytica and its influence on erythritol synthesis
Source: Microb Cell Fact. 2017 Jul 11;16:118. doi: 10.1186/s12934-017-0733-6 (PMC5504726; doi:10.1186/s12934-017-0733-6)
Supplement: Supplementary file 2 — Additional file 2: Figure S1. Quantification of genes expression belong to the ARK family, during erythritol synthesis (A). Quantification of YALI0F18590g gene expression (YlER,) by the strain AMM pAD-YIER and the control (B). Samples were analyzed in triplicate and the standard errors were estimated using Illumina Eco software. The results were normalized to actin gene ACT-F/ACT-R and analyzed using the ddCT method. [file 12934_2017_733_MOESM2_ESM.doc]

**Characterization of erythrose reductase from Yarrowia lipolytica and its influence on erythritol synthesis**

**Tomasz Janek^1^, Adam Dobrowolski^2^, Anna Biegalska^2^, Aleksandra M. Mirończuk^2*^**

**^1^**Department of Inorganic Chemistry, Faculty of Pharmacy, Wroclaw Medical University, Borowska 211a, 50-556 Wroclaw, Poland

**^2^**Department of Biotechnology and Food Microbiology, Wroclaw University of Environmental and Life Sciences, Chełmońskiego 37, 51-630, Wrocław, Poland

*corresponding author [aleksandra.mironczuk@upwr.edu.pl](mailto:aleksandra.mironczuk@upwr.edu.pl)


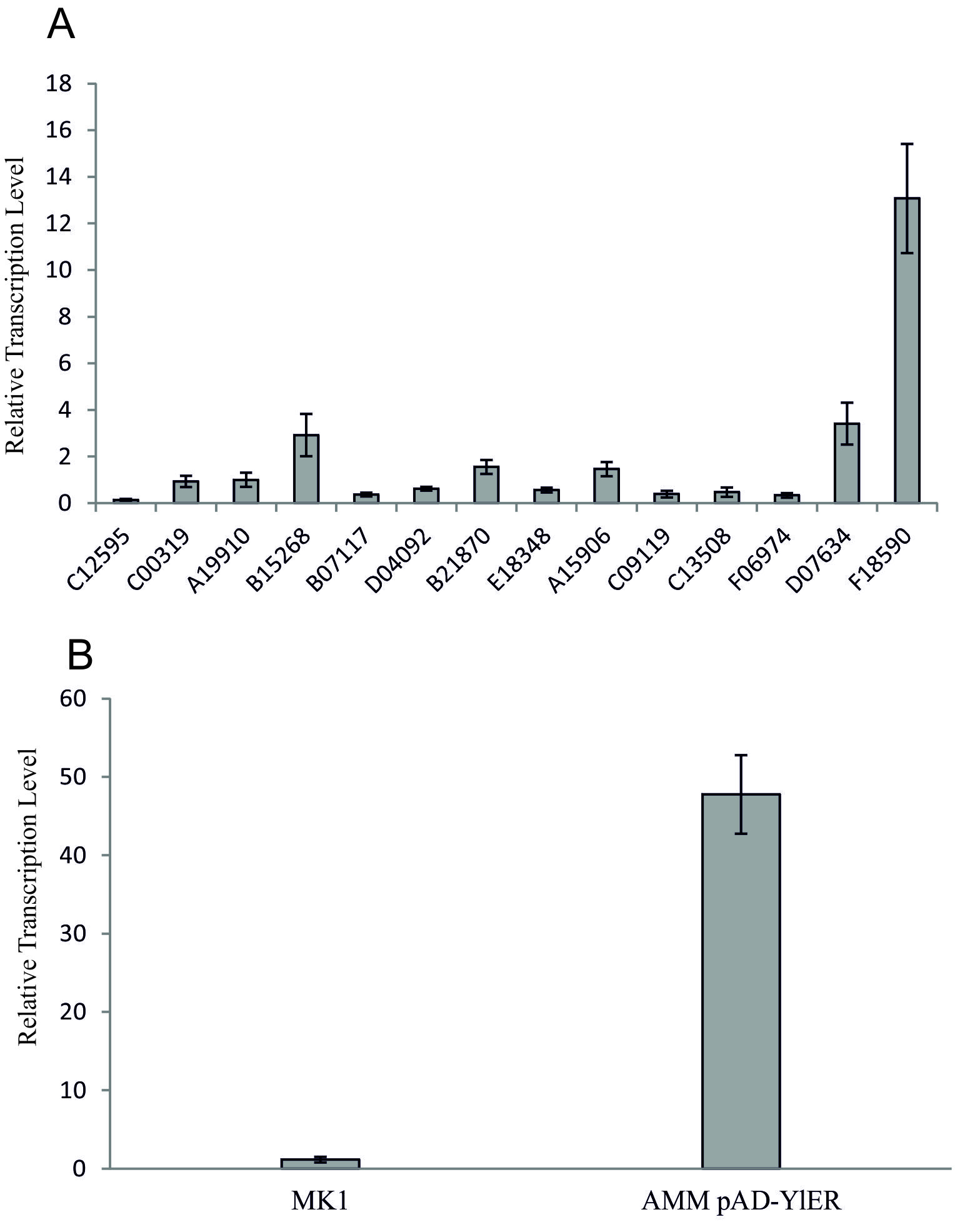


Figure S1. Quantification of genes expression belong to the ARK family, during erythritol synthesis (A). Quantification of *YALI0F18590g* gene expression (YlER,) by the strain AMM pAD-YIER and the control (B). Samples were analyzed in triplicate and the standard errors were estimated using Illumina Eco software. The results were normalized to actin gene ACT-F/ ACT-R and analyzed using the ddCT method.
