# Supplementary material for: Characterization of erythrose reductase from Yarrowia lipolytica and its influence on erythritol synthesis
Source: Microb Cell Fact. 2017 Jul 11;16:118. doi: 10.1186/s12934-017-0733-6 (PMC5504726; doi:10.1186/s12934-017-0733-6)
Supplement: Supplementary file 3 — Additional file 3: Figure S3. Erythritol synthesis by strain AMM ΔYlER (gray bars) and control strain MK1 (black bars). The cultures were performed in triplicate. The error bars represent the standard deviation. [file 12934_2017_733_MOESM3_ESM.doc]

**Characterization of erythrose reductase from *Yarrowia lipolytica* and its influence on erythritol synthesis**

**Tomasz Janek^1^, Adam Dobrowolski^2^, Anna Biegalska^2^, Aleksandra M. Mirończuk^2*^**

**^1^**Department of Inorganic Chemistry, Faculty of Pharmacy, Wroclaw Medical University, Borowska 211a, 50-556 Wroclaw, Poland

**^2^**Department of Biotechnology and Food Microbiology, Wroclaw University of Environmental and Life Sciences, Chełmońskiego 37, 51-630, Wrocław, Poland

*corresponding author [aleksandra.mironczuk@upwr.edu.pl](mailto:aleksandra.mironczuk@upwr.edu.pl)


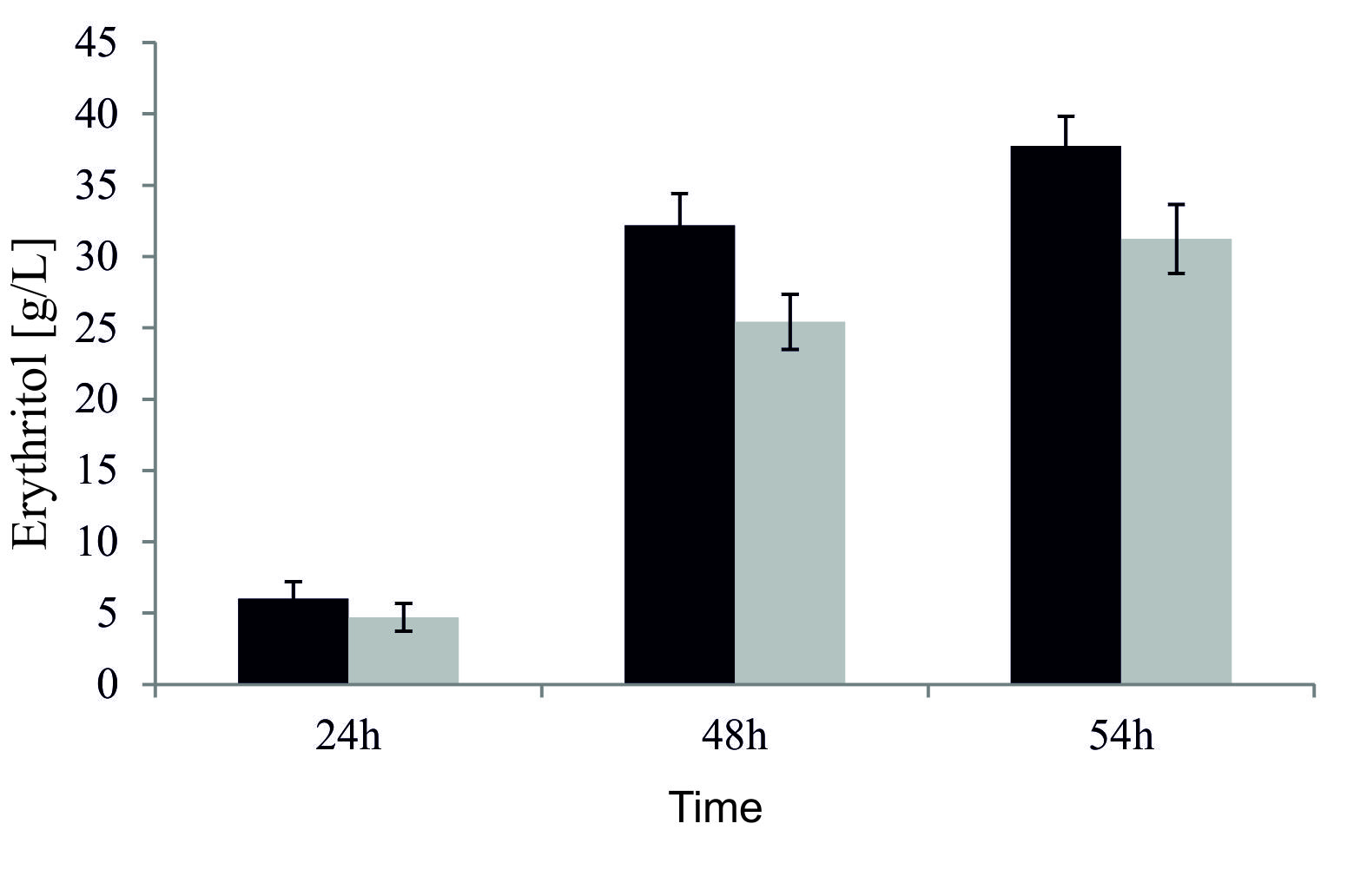


Figure S2. Erythritol synthesis by strain AMM ΔYlER (gray bars) and control strain MK1 (black bars). The cultures were performed in triplicate. The error bars represent the standard deviation.
