# Supplementary material for: Characterization of erythrose reductase from Yarrowia lipolytica and its influence on erythritol synthesis
Source: Microb Cell Fact. 2017 Jul 11;16:118. doi: 10.1186/s12934-017-0733-6 (PMC5504726; doi:10.1186/s12934-017-0733-6)
Supplement: Supplementary file 4 — Additional file 4: Table S2. Relative activity (%) of the erythrose reductases from Candida mangnoliae (CmER) [20], Yarrowia lipolytica (YlER), Moniliella megachiliensis (ER-III) [21] and Trichoderma reesei (Err1) [22]. [file 12934_2017_733_MOESM4_ESM.doc]

| **Substrate** | **CmER** | **YlER** | **ER-III** | **Err1** |
| --- | --- | --- | --- | --- |
| D-Arabinose | NT | 96 | 0 | 92^a^ |
| D-Erythrose | 100 | 100 | 100 | 100 |
| D-Fructose | 0 | 86 | 0 | NT |
| D-Galactose | 0 | 94 | 0 | NT |
| D-Glucose | 0 | 83 | 0 | 0 |
| D-glyceraldehyde | NT | NT | 66.0 | 118^b^ |
| D-Ribose | 5.6 | NT | 1.2 | NT |
| D-Xylose | 4.0 | NT | 1.2 | 0 |

**Characterization of erythrose reductase from *Yarrowia lipolytica* and its influence on erythritol synthesis**

**Tomasz Janek^1^, Adam Dobrowolski^2^, Anna Biegalska^2^, Aleksandra M. Mirończuk^2*^**

**^1^**Department of Inorganic Chemistry, Faculty of Pharmacy, Wroclaw Medical University, Borowska 211a, 50-556 Wroclaw, Poland

**^2^**Department of Biotechnology and Food Microbiology, Wroclaw University of Environmental and Life Sciences, Chełmońskiego 37, 51-630, Wrocław, Poland

*corresponding author [aleksandra.mironczuk@upwr.edu.pl](mailto:aleksandra.mironczuk@upwr.edu.pl)

Additional File 4: Table S2. Relative activity (%) of the erythrose reductases from *Candida mangnoliae* (CmER) [20], *Yarrowia lipolytica* (YlER), *Moniliella megachiliensis* (ER-III)[21] and *Trichoderma reesei* (Err1) [22].

^a^-L-arabinose used; ^b^- L- glyceraldehyde used; NT-not tested
